# Percutaneous Tracheostomy: A Bedside Procedure

**DOI:** 10.7759/cureus.24083

**Published:** 2022-04-12

**Authors:** Misbahuddin Khaja, Asim Haider, Anuhya Alapati, Zaheer A Qureshi, Laura Yapor

**Affiliations:** 1 Internal Medicine/Pulmonary and Critical Care, Icahn School of Medicine at Mount Sinai/Bronx Care Health System, New York City, USA; 2 Internal Medicine, Icahn School of Medicine at Mount Sinai/Bronx Care Health System, New York City, USA; 3 Pulmonary and Critical Care, Icahn School of Medicine at Mount Sinai/Bronx Care Health System, New York City, USA

**Keywords:** early tracheostomy, outcome of early versus late tracheostomy, tracheostomy procedure, tracheostomy complications, percutaneous tracheostomy

## Abstract

Percutaneous tracheostomy is a bedside surgical procedure that creates an opening in the anterior tracheal wall. Tracheostomy is performed in patients expected to require mechanical ventilation for longer than seven to 10 days. This bedside percutaneous tracheostomy has been used since the late 1990s. Tracheotomy tubes are of various kinds like cuffed vs. uncuffed, fenestrated vs. unfenestrated, single lumen vs. double lumen, and metal vs. plastic. Its indications are categorized into emergency vs. elective. The most common emergency indication is acute airway obstruction, and the elective indication is prolonged intubation. There is no absolute contraindication, but a physician should consider severe hypoxia requiring high oxygen and coagulopathy. Percutaneous tracheostomy is a new technique requiring different skills. Advantages of percutaneous tracheostomy are as follows - it is performed at the bedside, procedural time is less, the cost is less, does not need operating schedule time. Percutaneous tracheostomy is generally performed by otolaryngologists, general surgeons, interventional pulmonologists, thoracic surgeons, or intensivists.

## Introduction and background

Percutaneous tracheostomy is a bedside surgical procedure that creates an opening in the anterior tracheal wall. It is a life-saving procedure to maintain an airway when obstructed. It is performed in patients expected to require mechanical ventilation for longer than seven days, but the final decision is made on a case-by-case basis [1,2].

In the past tracheostomy procedure was known by different names laryngotomy, pharyngotomy, bronchotomy, and tracheotomy. Habicot in 1620 performed the first pediatric tracheotomy on a boy who swallowed a bag of gold that was obstructing the airway. In the literature, the word tracheotomy first appeared in 1649 but was officially introduced by Heisler, a German surgeon, in 1718. First, tracheostomies were done in children with airway inflammation for diphtheria in the early 1800s. Then, in 1808, the first successful reported case of tracheotomy was done in children [3].

## Review

There are two kinds of tracheostomy tubes, metal and nonmetal. Nonmetal tracheotomy tubes are of several types: cuffed vs. uncuffed, fenestrated vs. unfenestrated. Nonmetal tracheostomy tubes are cheaper, less rigid, adapt to the shape of the airway, and have an inflatable cuff and a universal adapter that connects to a ventilator. Sterilization is unnecessary since it is generally replaced every six to eight weeks. Metal tubes are not commonly used since they are expensive, rigid, does not have a cuff or common adapter to connect to a ventilator [4]. Cuffs tubes help in getting a tight seal while also limiting pressure on the tracheal wall (e.g., 20-30 cm H_2_O). However, complications with hyperinflated cuffs should be considered, like tracheomalacia, tracheal stenosis, fistula, and rarely necrosis of the tracheal wall causing perforation. Tracheostomy tubes can have a single cannula or dual cannula with easy removal and easy to clean inner cannula without taking out the tracheostomy tube from the site [5].

Tracheostomy can be open, done in the operating room or bedside. Percutaneous tracheostomy is done at the bedside using techniques like Ciaglia (Selinger technique), Griggs (forceps dilation), and Fantoni (translaryngeal). A tracheostomy provides passage for air when the usual breathing route is blocked. It bypasses the airway, which is obstructed, making it easy to clear secretions and deliver oxygen.

Percutaneous tracheostomy is generally performed by otolaryngologists, general surgeons, interventional pulmonologists, thoracic surgeons, or intensivists [6]. The percutaneous tracheostomy is advantageous because it is performed at the bedside, the time to perform is short, it is cost-effective, and the operating schedule is not needed. Also, complications like bleeding and infection are minimal with a percutaneous tracheostomy. The disadvantages of percutaneous tracheostomy are an increased risk of posterior tracheal wall injury, perforation, and the risk of anterior tracheal ring fractures [7,8]. The indications for tracheostomy are divided into emergency and elective indications [1,9,10].

Emergency indications

Emergency indications include (1) acute upper airway obstruction in which intubation is difficult like in angioedema, anaphylaxis, hematoma, retropharyngeal abscess, or a mass malignant or benign tumors compressing airway, and foreign body; (2) fractures of the nose, facial bones, and neck; (3) laryngeal injury from trauma; and (4) burns.

Main elective indications

There are three main elective indications. Firstly, prolonged intubation or ventilator dependence - an elective tracheostomy provides long-term ventilation and facilitates weaning from the ventilator. It is a more stable airway than an endotracheal tube, avoids complications of long-term intubation, improves patient comfort, decreases dead space, and extubation trials are easier. Secondly, mechanical airway obstruction - the conditions of mechanical airway obstruction that benefit from elective tracheostomy are congenital (including subglottic stenosis, webs, and cysts), trauma (e.g., due to a foreign body, burns, and corrosive), and infectious conditions (e.g., epiglottitis, Ludwig’s angina, and croup). Also, disorders like vocal cord paralysis and obstructive sleep apnea. And thirdly, inability of a patient to manage secretions - the conditions where the patient is unable to clear or manage their secretions include neurological diseases (e.g., bulbar palsy, multiple sclerosis, and myasthenia gravis), coma due to head injury, stroke, drug overdose, and head/neck surgery.

Indications for an open surgical approach

Indications for an open surgical approach are as follows - (a) a short neck with the inability to palpate and identify the trachea; (b) inability to extend the neck because of cervical trauma, cervical instability, cervical fusion, and history of rheumatoid arthritis; (c) patients requiring emergency airways, children less than 15 years; and (d) conditions like high-riding innominate or thyroid internal mammary artery, neck with scarring, hematoma, thyromegaly, large extensive tumor, severe tracheomalacia, and cartilage destruction [11].

Contraindication

There are no absolute contraindications, but a few clinical conditions should be considered - (a) severe hypoxemia whose fraction of inspired oxygen (FiO_2_) requirement is more than 60% and positive end-expiratory pressure (PEEP) of >12 cm H_2_O; (b) uncorrectable bleeding diathesis (e.g., international normalized ratio >2.0, platelets <50,000 x 10^9^/L); and (c) hemodynamic instability requiring pressor support [12].

Advantages of tracheostomy compared to oral intubation

The most common advantages of tracheostomy over oral intubation are as follows - (a) more secure airway, increased comfort, phonation, communication, increased patient mobility, improved airway suctioning, less direct endolaryngeal injury, and enhanced oral nutrition; (b) early transfer of the ventilator-dependent patient from the intensive care unit and decreased airway resistance help in weaning [13,14].

Considerations regarding anticoagulation for percutaneous tracheostomy

Some special considerations regarding the continuation vs. discontinuation of various forms of anticoagulants prior to percutaneous tracheostomy are as follows - aspirin and deep venous thrombosis (DVT) prophylaxis should be continued. Coumadin should be held until the international normalized ratio is less than 1.5 or reverses with fresh frozen plasma. The novel oral anticoagulants (NOACs) should be held between 24 and 48 hours. Hold clopidogrel for five days before the procedure. If the patient is on extracorporeal membrane oxygenation, heparin should be held for four hours before and two hours postprocedure in agreement with intensive care unit (ICU) teams and extracorporeal membrane oxygenation team [15,16].

Percutaneous tracheostomy kit

Percutaneous tracheostomy kits are pre-prepared kits that contain a sterile drape, syringe, lidocaine 1% local anesthetic, introducer needle, guidewire, tracheal dilators, and tracheal loading trocar [17]. We need to have an airway backup plan in case there is a loss of airway while doing the procedure, which includes having an artificial manual breathing unit (Ambu; Hythe, UK: Smiths Medical International Ltd.) bag connect to an oxygen port, a suction device, and an airway cart with an endotracheal tube with GlideScope (Bothell, WA: Verathon Medical Inc.).

Procedure technique

Bronchoscopic guided percutaneous tracheostomy via the Ciaglia technique is safe with fewer complications. Two physicians are required to perform bronchoscopy with an endotracheal tube and perform a percutaneous procedure at the neck. In addition, we need a nurse to give sedation medications, monitor blood pressure every two minutes, a telemetry monitor along with a pulse oximeter, and a respiratory therapist to manage the ventilator.

After time-out is performed, the patient is positioned with neck extended by placing shoulder roll beneath scapula. Ultrasound is used to locate high innominate artery before the procedure. Then start cleaning the neck area with chlorhexidine and cover with a sterile drape to expose only the neck area. Connect the bronchoscopy adaptor to the endotracheal tube and inspect the airway with the help of a bronchoscope. Then the tracheal lumen is fully visualized. The endotracheal tube's cuff is deflated, and the respiratory therapist starts pulling the endotracheal tube to the proximal end of the trachea, which is a subglottic area, then the cuff can be reinflated. The surgeon can identify the landmark by using transillumination of bronchoscope light.

Local anesthesia with lidocaine is given using a 25-gauge needle, which the bronchoscopy can visualize. Then the introducer needle is inserted perpendicular to the anterior wall of the trachea between the second and third cartilage rings and once visualized with a bronchoscope. The needle is angled caudally, leaving a plastic catheter and removing the needle. Then the guidewire is passed through the plastic catheter, which should be visualized with a bronchoscope going towards the carina. The introducer catheter is removed, and a vertical midline incision of 1.5-2 cm is performed on the skin of the trachea by scalpel. The small punch dilator is passed over the guidewire using the Seldinger technique, and then the dilator is removed with the guidewire still in place. Then the blue rhino single-stage progressive dilator is introduced over the guidewire to dilate the tract using the Seldinger technique. The dilator is removed, keeping the guide catheter and wire in place. The loading dilator is inserted in the tracheostomy and advanced in the trachea using the guide catheter and guidewire. The loading dilator, guide catheter, and guidewire are removed.

The percutaneous tracheostomy is in place, and the cuff is inflated. Then perform bronchoscopy through the new tracheostomy to confirm its position in the carina. Then ventilator is disconnected from the endotracheal tube and connected to a tracheostomy. We recommend further use of end-tidal CO_2_ to verify its position and check the return of tidal volume on a ventilator. Next, the tracheostomy tube is secured with soft ties collar. To prevent ulceration underneath the tracheal site, we prefer to place sterile gauze underneath the flange. Finally, the endotracheal tube cuff is deflated and removed [18].

The physician should remember that maintaining a bronchoscope during a percutaneous tracheostomy procedure can lead to hypoventilation, respiratory acidosis, and hypercarbia. On the other hand, the ultrasound technique to localize the endotracheal tube reduces hypercarbia. Therefore, to minimize this small bronchoscope and shorten the time to perform percutaneous tracheostomy is preferred [19].

Complications 

Complications range between 5% and 40%, with an average mortality of 2%. Complications are more frequent in emergencies, severely ill patients, and children and are categorized as early (intraoperative, immediate postoperative) vs. late complications [6,11,20].

Intraoperative complications

Hemorrhage

The chances of intraoperative (or immediate postoperative) hemorrhage are 5%. The most common sites of hemorrhage include anterior jugular veins, thyroid isthmus, high Innominate artery, and thyroid ima artery.

Pneumothorax-Pneumomediastinum

It accounts for 2-5% of the adult population and 17% of the pediatric population. The generally accepted mechanisms include forceful inspiration leading to high negative intrathoracic pressure, direct injury to apical pleura (especially in children due to high position), rupture of lung bleb, and surgical technique/error.

Other Intraoperative Complications 

Other intraoperative complications include recurrent laryngeal nerve injury, inability to identify trachea, and tube misplacement (into bronchus or outside tracheal lumen). One of the most common causes of operating room fires is electrocautery when using high oxygen concentrations (50%).

Postoperative complications

Early

Early postoperative complications include massive bleeding from vessel erosion, infection, pneumothorax, subcutaneous emphysema, pneumomediastinum, malpositioned tube, and obstruction.

Late or Delayed

Late postoperative complications include displacement/false passage, mucus plug, tracheocutaneous fistula, tracheoesophageal fistula, tracheal stenosis, tracheomalacia, dysphagia, and tracheal damage. Direct pressure necrosis can occur from the cuff or tip of the tube; cuff pressure should not exceed 30 mmHg. Capillary flow should not exceed 30-50 mmHg. Mucosal ulceration causes cartilage exposure leading to bacterial colonization, stricture, and malacia. Excessive traction on tracheostomy tube by connecting tubing or patient motion leads to skin necrosis.

Early vs. late tracheostomy

Many prospective studies are well designed, but it is difficult to power a prospective study that would pick up small changes between early and late tracheostomy groups. When analyzing the data, some studies suggest benefits for early tracheotomy, and some do not. Many studies find benefits for some aspects of early tracheostomy (such as increased comfort or incidence of ventilator-associated pneumonia {VAP}) but not for others [21,22].

The study done by Griffiths et al. showed that early tracheostomy did not alter mortality and risk of pneumonia but decreased the duration of mechanical ventilator and intensive care unit length of stay [23]. Prospective analysis done by Rumbak et al. showed that the early tracheostomy group had less mortality, pneumonia (5% vs. 25%), less unplanned extubation, fewer days in the intensive care unit, and mechanical ventilator compared to the prolonged intubation group [24]. In neurosurgical patients with poor Glasgow Coma Scores, early tracheostomy was associated with a decreased colonization rate of pathogens in the tracheobronchial tree, faster weaning from the ventilator, and improved lung infections [25].

A study done by Silva et al. showed that the early group was associated with a decreased sedation requirement [26]. The meta-analysis done by Wang et al. showed that early tracheostomy is associated with a lower incidence of ventilator-associated pneumonia, the requirement of sedation was less, decreased intensive care unit length of stay, and decreased duration of mechanical ventilators [27]. There is currently no information about early or late tracheostomy having a better outcome. Therefore, the conclusion is that more randomized trials are needed to focus on outcomes.

## Conclusions

Percutaneous tracheostomy is a bedside procedure that is safe to perform, has less procedural time, has low cost, and does not need operating schedule time. The most common intra- and postoperative complication is bleeding. Access to ultrasound to locate a high innominate artery can prevent the sentinel bleeding complication. Percutaneous tracheostomy can be performed by thoracic surgeons or intensivists, otolaryngologists, general surgeons, and interventional pulmonologists.
